# Toll-Like Receptor 2 Signaling Protects Mice from Tumor Development in a Mouse Model of Colitis-Induced Cancer

**DOI:** 10.1371/journal.pone.0013027

**Published:** 2010-09-27

**Authors:** Emily L. Lowe, Timothy R. Crother, Shervin Rabizadeh, Bing Hu, Hanlin Wang, Shuang Chen, Kenichi Shimada, Michelle H. Wong, Kathrin S. Michelsen, Moshe Arditi

**Affiliations:** 1 Division of Pediatric Infectious Diseases and Immunology, Cedars-Sinai Medical Center and David Geffen School of Medicine at University of California Los Angeles, Los Angeles, California, United States of America; 2 Division of Pediatric Gastroenterology, Cedars-Sinai Medical Center and David Geffen School of Medicine at University of California Los Angeles, Los Angeles, California, United States of America; 3 Department of Pathology, Cedars-Sinai Medical Center and David Geffen School of Medicine at University of California Los Angeles, Los Angeles, California, United States of America; 4 Inflammatory Bowel Disease Center & Immunobiology Research Institute, Burns and Allen Research Institute, Cedars-Sinai Medical Center and David Geffen School of Medicine at University of California Los Angeles, Los Angeles, California, United States of America; University of California Los Angeles, United States of America

## Abstract

Inflammatory bowel disease (IBD) is a disorder of chronic inflammation with increased susceptibility to colorectal cancer. The etiology of IBD is unclear but thought to result from a dysregulated adaptive and innate immune response to microbial products in a genetically susceptible host. Toll-like receptor (TLR) signaling induced by intestinal commensal bacteria plays a crucial role in maintaining intestinal homeostasis, innate immunity and the enhancement of intestinal epithelial cell (IEC) integrity. However, the role of TLR2 in the development of colorectal cancer has not been studied. We utilized the AOM-DSS model for colitis-associated colorectal cancer (CAC) in wild type (WT) and TLR2^−/−^ mice. Colons harvested from WT and TLR2^−/−^ mice were used for histopathology, immunohistochemistry, immunofluorescence and cytokine analysis. Mice deficient in TLR2 developed significantly more and larger colorectal tumors than their WT controls. We provide evidence that colonic epithelium of TLR2^−/−^ mice have altered immune responses and dysregulated proliferation under steady-state conditions and during colitis, which lead to inflammatory growth signals and predisposition to accelerated neoplastic growth. At the earliest time-points assessed, TLR2^−/−^ colons exhibited a significant increase in aberrant crypt foci (ACF), resulting in tumors that developed earlier and grew larger. In addition, the intestinal microenvironment revealed significantly higher levels of IL-6 and IL-17A concomitant with increased phospho-STAT3 within ACF. These observations indicate that in colitis, TLR2 plays a protective role against the development of CAC.

## Introduction

Chronic inflammation and cancer are intertwined, especially in the intestine, as seen in inflammatory bowel disease (IBD). IBD is thought to result from a breakdown at the epithelial barrier, followed by inappropriate responses to microbial products resulting in chronic inflammation in a genetically susceptible host [1]. IBD is associated with an increased risk for colorectal cancer and it is now commonly believed that the chronic inflammation in these patients leads to the neoplastic transformation of intestinal epithelium [2]. Proper intestinal immunity relies on a balance between immunosuppression and appropriately timed proinflammatory responses with protective inflammatory responses. Proinflammatory cytokines and chemokines that are produced during chronic intestinal inflammation in response to commensal bacteria create a microenvironment that enhances cell proliferation, cell survival, and angiogenesis, thereby promoting tumorigenesis [3].

Intestinal epithelial cells (IEC) act as the first line of defense by creating a barrier against microbes. The innate immune receptors belonging to the family of Toll-like receptors (TLR) further help IEC to distinguish friend from foe among the complicated milieu of microbes. There is a growing body of evidence to support a significant role for TLR signaling in the maintenance of intestinal homeostasis [4], [5], [6], [7], [8], [9]. Mice ablated of TLR2, TLR4, TLR9, or their common adaptor molecule MyD88, were more acutely susceptible to colitis induced by the chemical colitogen dextran sodium sulfate (DSS), due in part to impaired protective responses and reduced prostaglandin production [7], [8], [9], [10], [11]. Antibiotic treatment worsened DSS-induced colitis in MyD88^−/−^ mice and prevented epithelial repair, indicating general TLR signaling is required for proper IEC renewal. Indeed, treatment with TLR2 [8] and TLR9 [12] ligands during DSS administration ameliorated crypt damage and sped healing. TLR2-commensal signaling preserves transepithelial resistance, promotes goblet cell mucin secretion, and maintains homeostasis within IEC [8], [13], [14], [15]. Finally, because of its tendencies to skew towards TH2 responses [16], [17], [18], [19] and enhance regulatory T cell survival [20], [21], [22], TLR2 signaling might play a major role in maintaining a suppressive environment in the colon.

Various studies have implicated TLR signaling in intestinal tumorigenesis. In multiple intestinal neoplasia (Min) mice, loss of MyD88 signaling reduced tumor numbers and sizes suggesting that MyD88 signaling contributes to tumor growth and progression [23]. In a different model, tumor incidence, multiplicity and size were reduced after azoxymethane (AOM) and DSS treatment when TLR4 was ablated in mice via reduction of TLR4 expression and signaling in IEC [24], [25]. In addition, the colitis and colitis-associated colorectal tumors (CAC) spontaneously generated in IL10^−/−^ mice can be ameliorated if mice are kept in germ-free conditions [26] or concurrently ablated of TLR4 [27]. Taken together, these studies support that CAC development depends on intestinal TLR recognition of commensal bacteria. Despite the evident roles of TLR2 in IEC homeostasis and enhancement of tolerance in the lamina propria microenvironment [28], [29], no study has elucidated the role that TLR2 might play in transformation of intestinal epithelial cells leading to intestinal tumors.

Here we demonstrate that TLR2-deficient mice develop more and larger colonic tumors than WT control mice after AOM-DSS treatment. Enhanced colonic tumor development is evidenced early on by significantly increased numbers of aberrant crypt foci (ACF) and increased IL-6, IL-17A, TNFα and phosphoSTAT3 expression in the intestinal microenvironment of TLR2^−/−^ mice as compared to WT mice. Our results demonstrate that TLR2 is an important protective factor in intestinal epithelial homeostasis and provide important insights into a previously unrecognized role of TLR2 signaling in CAC.

## Materials and Methods

### Ethics Statement

All experiments were performed according to the guidelines and approved protocols (IACUC #2838) of the Cedars-Sinai Medical Center Institutional Animal Care and Use Committee and were housed under specific pathogen free conditions.

### Animals

Helicobacter-negative wild-type C57BL/6J and TLR2^−/−^ (on C57BL/6J background) mice were obtained from the Jackson Laboratory (Bar Harbor, ME).

### Induction of tumors (figure 1A)

Colitis-associated colorectal cancer (CAC) was induced as previously described [30]. Briefly, 6–8 week old mice were injected intraperitoneally (IP) with 12.5 mg/kg azoxymethane (AOM; Sigma-Aldrich Chemical Co, St. Louis, MO). After 5 days, mice received 2.5% dextran sulfate sodium (DSS; MP Biomedicals, Solon, OH, molecular weight 35,000–50,000 kDa) water for 5 days followed by 16 days of regular drinking water. Mice were subjected to two DSS cycles, followed by a third cycle with 2% DSS water administered for 4 days followed by regular water for 10 days. On day 61 post AOM injection mice were injected IP with 100 mg/kg 5-bromo-2-deoxyuridine (BrdU; Sigma-Aldrich Chemical Co, St. Louis, MO) and sacrificed 2.5 h later. To observe the earliest transformative steps in CAC, mice were sacrificed 4 days after the completion of the first DSS cycle (14 days post AOM). The clinical course of disease was monitored by measurement of body weight, observation of rectal bleeding, diarrhea, and bloody stool during DSS treatment.

**Figure 1:**
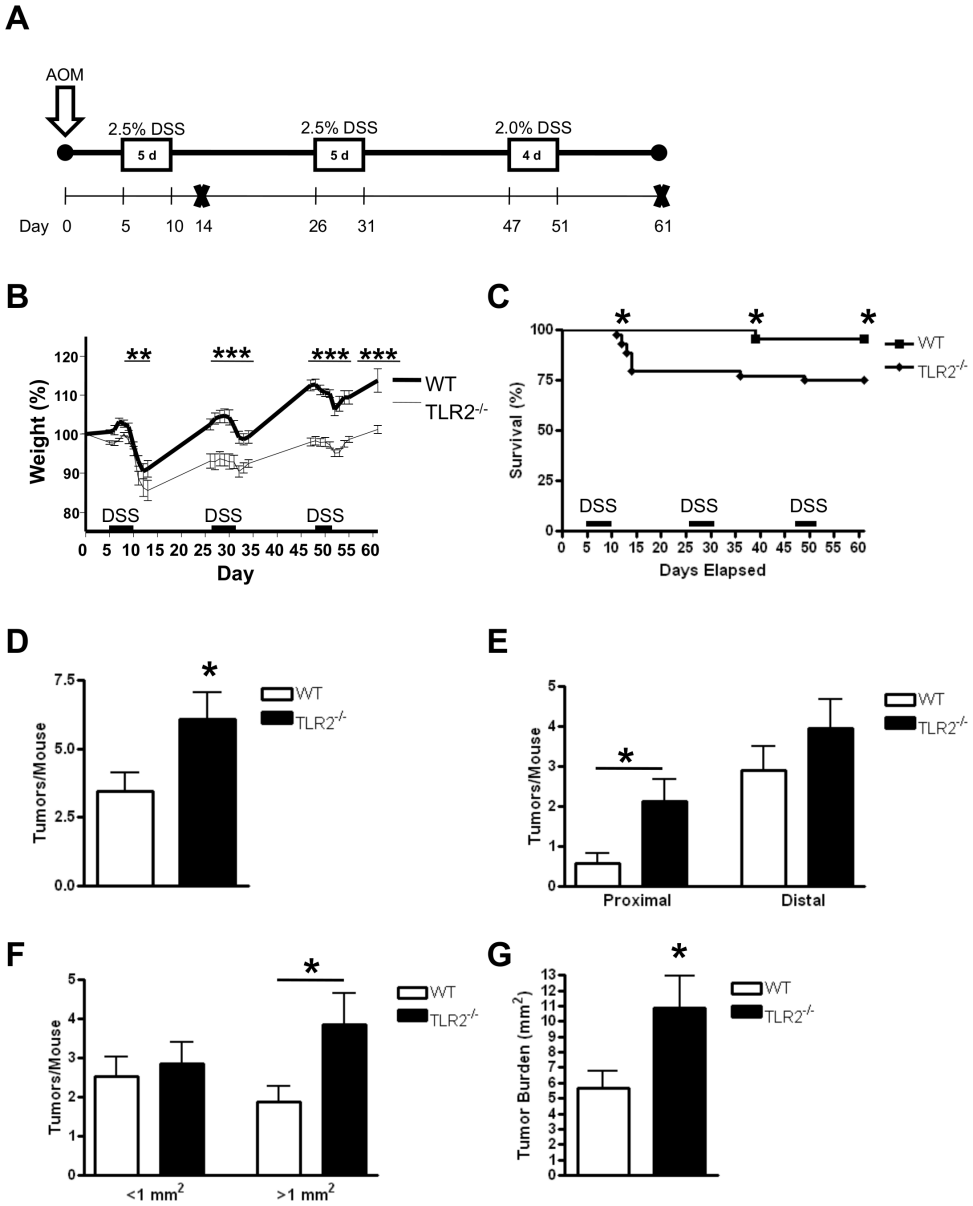
TLR2-deficiency leads to increased development of colitis-associated colon cancer. (A) Schematic overview of the CAC model. After initial AOM injection (12.5 mg/kg), DSS was given in the drinking water (boxed areas) followed by regular drinking water. Mice were sacrificed on days 14 or 61 post AOM injection (Day 61: n = 19 WT, n = 21 TLR2^−/−^ mice). (B) Percent weight change during AOM-DSS treatment. (C) Mouse mortality during AOM-DSS treatments. (D) Number of colorectal tumors per mouse induced by AOM-DSS treatment at day 61. (E) Number of tumors per mouse located in proximal or distal colons in WT or TLR2^−/−^ mice. (F) Size distribution of colorectal tumors formed in WT or TLR2^−/−^ mice. (G) Tumor burden in AOM-DSS treated WT or TLR2^−/−^ mice. All tests were performed using 95% confidence intervals. Data are expressed as means ± SEM. * = p<0.05, ** = p<0.01, *** = p<0.001.

### Histological Analysis for Colitis and Dysplasia

Entire colons were flushed with PBS and flash-frozen in the swiss roll orientation. 7-µm sections were collected throughout the depth of the colon using a cryostat and stained for hematoxylin and eosin (H&E: Sigma-Aldrich). Histopathological alterations in colitis and inflammation were scored by a Pathologist (HW) blinded to the genotypes using the following scoring systems. Data are presented in results section as “overall inflammation score”, which represents the sum of the scores given for “inflammation”, “extent and severity of leukocyte infiltration” and “percent involvement of the colon”. The definition and scoring for these subcriterias are as follows: *Inflammation was defined and scored as*: (0) None to normal lymphoid aggregates; (1) Increased lymphoid aggregates; (2) Cryptitis (neutrophils within crypt epithelium); (3) Crypt abscess (neutrophils accumulating within crypt lumen, sometimes with crypt rupture); (4) Ulceration (loss of mucosal components and presence of granulation tissue). *Extent of leukocyte infiltration was scored as*: (0) None; (1) infiltration confined to the mucosa; (2) infiltration extending to the submucosa; (3) Transmural extension of inflitration. *Severity of leukocyte infiltration was scored based on number of infiltrating cells as*: (1) Mild; (2) Moderate; (3) Severe. *Percent Involvement of colon was scored as*: (0.25) if 1–25% of the colon was involved; (0.50) if 26–50% of the colon was involved; (0.75) if 51–75% of the colon was involved; (1.00) if 76–100% of the colon was involved. Separately, we have also scored the “*extent necrosis*” in the colon as follows: (1) = 25%, (2) = 50%, (3) = 75%, (4) = 100%. Neoplasms were identified and also scored for severity (adenoma or carcinoma *in situ*). Tumor burden in mice was determined by the sum of the areas of all tumors per mouse.

### BrdU and TUNEL Staining

All neoplasms observed by H&E were confirmed after BrdU staining using BrdU *In-Situ* Detection Kit (BD Pharmingen, San Diego, CA) according to manufacturer's suggested protocol. Apoptosis was detected using the *In Situ* Cell Death Detection Fluorescein Kit (Roche, Indianapolis, IN), counterstained with DAPI (Invitrogen, Carlsbad, CA) and quantified by fluorescence intensity per focus field using ImagePro Plus (Media Cybernetics, Silver Spring, MD). Proximal and distal regions of colon were examined using greater than three focus fields per region in at least four slides per animal.

### Immunofluorescence

Frozen sections were fixed in 10% formalin. Slides stained for nuclear antigens were further fixed in 100% methanol. Anti-phospho-Stat3 and anti-ß-catenin (both from Cell Signaling Technology, Danvers, MA) antibodies were incubated for 48 h at 4°C followed by incubation with goat anti-rabbit-568 (Invitrogen, Carlsbad, CA). Biotinylated anti-nitrotyrosine (Cayman, Ann Arbor, MI) was incubated overnight at 4°C followed by incubation with streptavidin-594 (Invitrogen, Carlsbad, CA). All slides were counterstained with DAPI (Invitrogen, Carlsbad, CA). Positive cells were counted in greater than five focus fields in at least four slides per animal and nuclear phospho-Stat3 intensity was measured using ImagePro Plus.

### Isolation and Treatment of Lamina Propria Mononuclear Cells (LPMC) and Mesenteric Lymph Node Cells (MLN)

To obtain LP cells, entire colons were thoroughly flushed with ice-cold PBS. One-mm pieces of colon were incubated in HBSS supplemented with 1 mM DTT, 3 mM EDTA, 20 mM HEPES with shaking at 37°C for 20 min. Colon pieces were washed with HBSS followed by incubation in dissociation buffer (0.075 U/ml Blendzyme 3, 1.5 U/ml Dispase II, 0.5 mg/ml DNase I [all Roche Diagnostics, Mannheim, Germany], 20 mM HEPES, 1.3 mM calcium chloride) with shaking at 37°C for 50 min. The digested colons were vortexed briefly and further dissociated with increasing gauge needles, passed through a 40 µm cell strainer and washed with PBS. After centrifugation the cell pellet was resuspended in a 45% Percoll solution and laid over a 72% Percoll solution. Cells located at the interphase were collected in a clean tube and washed several times. MLN were crushed between glass slides and washed with PBS. The cell suspension was passed through a 40 µm cell strainer and washed with RPMI. Isolated lymphocytes were resuspended in 10% FBS-high glucose RPMI supplemented with L-glutamine (Cellgro; Mediatech Inc., Herndon, VA) and 1% antibiotic/antimycotic (Sigma-Aldrich Chemical Co, St. Louis, MO), and stimulated with 1 µg/ml anti-CD3 (eBioscience, San Diego, CA) and 1 µg/ml anti-CD28 (eBioscience) for 72 h or with 1 µg/ml LPS (InvivoGen, San Diego, CA) for 6 h. Supernatants were collected and stored at −80°C.

### Preparation of Colon Homogenates

Colons were flushed thoroughly with ice-cold PBS. Colons were then flash-frozen in liquid nitrogen. Washed and sterilized zirconium oxide beads (0.5 mm diameter, Next Advance, Cambridge, MA) were added in a volume equal to that of the tissue. Colon tissues were then briefly mechanically homogenized using the Bullet Blender (Next Advance, Cambridge, MA) at 4°C. An isotonic lysis buffer (1 mM EDTA, 50 mM HEPES-NaOH, pH 7.9, 250 mM NaCl, 20 mM ß-glycerophosphate, 1 mM activated orthovanadate, 1% NP-40, 1 mM DTT) was then added to the tissue and beads in a volume equal to that of the tissue. This was further homogenized followed by rotation at 4°C for 20 min. Homogenized tissues were centrifuged for 20 min at 4°C and 16,100 *g* and supernatants were stored at −80°C.

### ELISAs

Supernatants, colon homogenates or serum were analyzed for the presence of TNFa, IL-4, IFNg, IL-17A, IL-23p19 (all eBioscience, San Diego, CA), IL-10, IL-6, TGFß, MIP-2 (all R&D Systems, Minneapolis, MN), or KC, MCP-1 and RANTES (all BD Biosciences, San Jose, CA), as per the manufacturer's suggested protocol.

### Statistical analysis

Statistical analyses were performed using the software GraphPad Prism 4. Statistical analysis of the survival curves was performed using log-rank test. Statistical analyses of tumor sizes were performed using the Mann-Whitney test for populations not following Gaussian distributions. Comparisons of one-variable data following Gaussian distributions were performed using a two-tailed unpaired Student's T-test. When an F-test indicated variances differed significantly, Welch's correction to the Student's T-test was employed. Comparisons of two variable data were performed using two-way ANOVA with Bonferroni post test. Comparisons of survival curve data were performed using the log-rank (Mantel-Cox) test. All tests were performed using 95% confidence intervals. Data are expressed as means ± SEM. * = p<0.05, ** = p<0.01, *** = p<0.001.

## Results

### TLR2-deficiency leads to increased development of colitis-associated colon cancer

Previous studies have shown that TLR signaling and the common adaptor molecule MyD88 are critically involved in intestinal epithelial cell homeostasis and the development of intestinal tumors [9], [10], [23], [24], yet the role of TLR2 has not been extensively studied. To examine the role of TLR2 during colitis-associated tumorigenesis we used a well established model of CAC [4], [24], [30], [31], [32]. WT and TLR2^−/−^ mice received a single injection of AOM followed by administration of three cycles of DSS (Figure 1A). TLR2^−/−^ mice had increased morbidity as evidenced by increased weight loss during the course of treatment (Figure 1B) and more rectal bleeding during DSS treatment as compared to WT mice (data not shown). We also found increased mortality in the TLR2−/− mice compared to wild type mice (4.6% for WT and 25% for TLR2^−/−^ mice, respectively, p<0.05), with most deaths occurring shortly after the first course of DSS (Fig 1C). Next, we examined the impact of TLR2-deficiency on colitis-associated tumor development. TLR2^−/−^ mice had a higher tumor burden than WT mice. Histolopathological examination revealed a significant increase in the numbers of tumors in TLR2^−/−^ compared to WT mice (Figure 1D). The average tumor number per mouse was almost doubled in TLR2-deficient mice compared to WT mice (6.1 vs. 3.5, p<0.05). Because intestinal TLR2 is more strongly expressed in the proximal colon [33], we hypothesized that mice lacking TLR2 would develop more CAC proximally. Indeed, the difference in tumor development in TLR2^−/−^ compared to WT mice was most prominent in the proximal colon (Figure 1E). In addition to the increase in tumor multiplicity, TLR2-deficiency also led to significantly higher number of larger tumors (>1 mm^2^) (p<0.05) (Figure 1F) and higher tumor burden (nearly double) in TLR2^−/−^ mice compared to WT mice (p<0.05) (Figure 1G). These results indicate that TLR2-deficiency not only enhances tumor promotion but also increases tumor progression.

### TLR2-deficient colons have more advanced dysplasia and ß-catenin expression compared to WT colons

**Figure 2:**
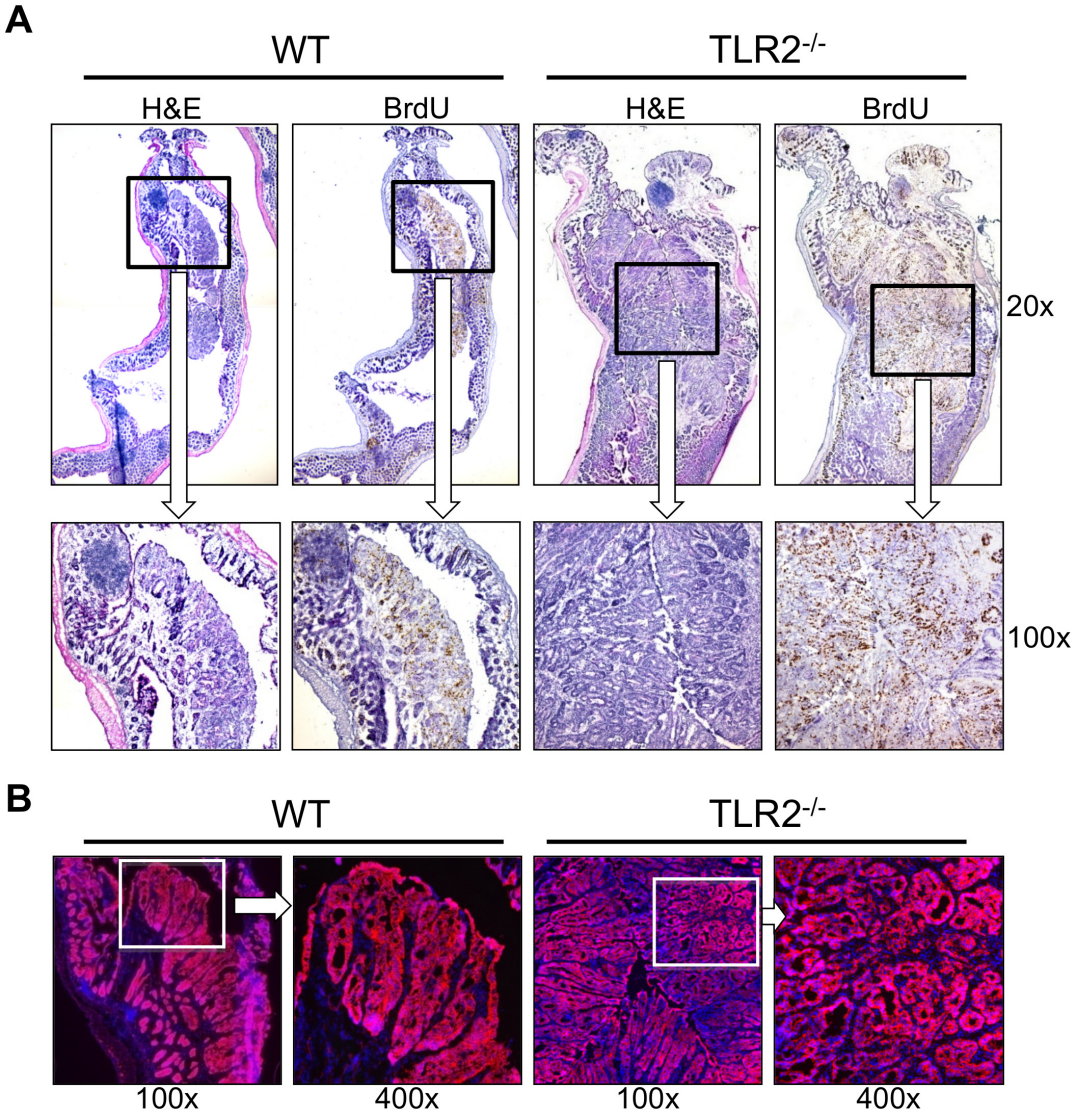
TLR2-deficient colons have more advanced dysplasia and ß-catenin expression compared to WT colons. Histopathology of colons at day 61 of AOM-DSS treatment. (A) Hematoxylin and eosin (H&E) (left) or BrdU stained (right) serial sections of WT or TLR2^−/−^ mice are shown. Original magnification 20x (upper panels) and 100x (lower panels) are shown. (B) Immunofluorescent staining for ß-catenin. Original magnification 100x (left panel) or 400x (right panel).

ß-catenin signaling plays an essential role in human intestinal carcinogenesis [34] and mutations have been reported in AOM-induced murine colonic tumors [30], [31], [35]. Indeed, strongly positive cytoplasmic or nuclear ß-catenin staining was observed in transformed tissue in WT and TLR2^−/−^ mice (Figure 2B). WT colorectal adenomas (Figure 2A, B, left 2 panels) displayed classic tubular structures with distorted glands and moderate organization. However, TLR2^−/−^ colorectal neoplasms (Figure 2A, B, right 2 panels) displayed more distorted glands with regions of increased disorganization and cribriform structures indicative of a higher grade dysplasia (i.e. carcinoma *in situ)*. In fact, histological analysis revealed almost twice the incidence of advanced carcinoma *in situ* (0.3 vs 0.6 mean carcinoma in situ per mouse) in TLR2^−/−^mice as compared to WT, which trended towards significance (p<0.055) (Figure S1).

### TLR2-deficiency leads to increased early formation of aberrant crypt foci and early intestinal tumorigenesis

**Figure 3:**
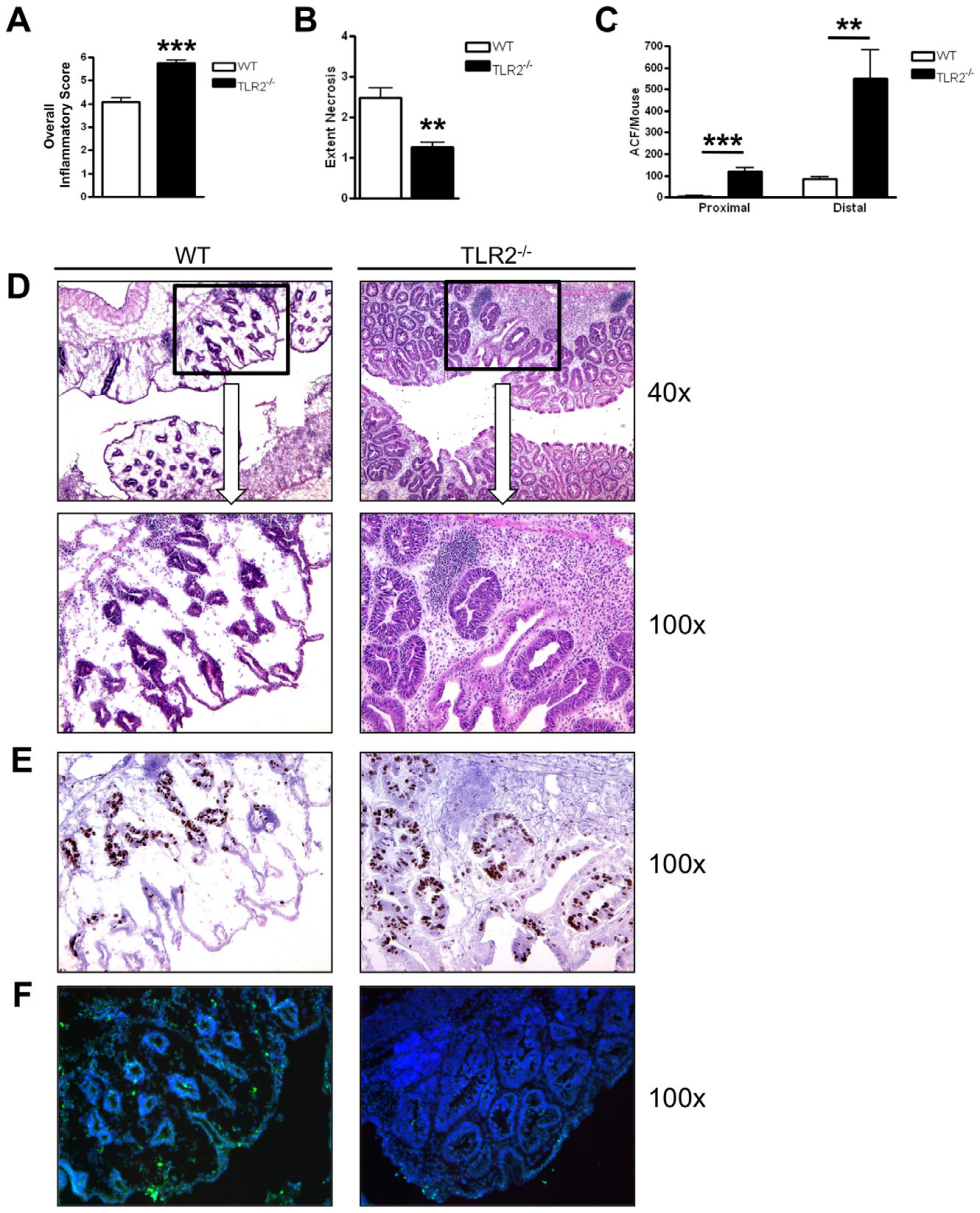
TLR2-deficiency leads to early formation of aberrant crypt foci. Scoring of inflammation, necrosis and ACF at day 14 of AOM-DSS treatment (n = 5 WT and n = 5 TLR2^−/−^). (A) Inflammatory scores of colons. (B) Extent of colonic necrosis. (C) Number of ACF per mouse located in proximal or distal colons. (D) H&E stains of serial sections of colons. Original magnification 40x (upper panel) or 100x (lower panel). (E) Immunohistochemical stains for BrdU. Original magnification 100x. (F) Immunofluorescent TUNEL staining. Original magnification 100x. All tests were performed using 95% confidence intervals. Data are expressed as means ± SEM. * = p<0.05, ** = p<0.01, *** = p<0.001.

Because TLR2^−/−^ mice developed significantly larger colonic tumors, we next examined WT and TLR2 deficient colons 14 days into the AOM-DSS regimen for the earliest transformative events during tumorigenesis. At this time point, TLR2^−/−^ mice showed significantly increased inflammation compared to WT mice (overall inflammation score of 4 vs 6, p<0.001) (Figure 3A). Colons from TLR2^−/−^ mice contained moderate to severe inflammation that extended transmurally while inflammation in WT colons extended to similar depths but displayed only mild to moderate infiltration of leukocytes (Figure 3D). The increased inflammation found at this time point (day 14) in TLR2−/− mice also correlated with the more severe body weight loss and increased mortality during the first course of DSS in our long term study (Fig 1B–C). WT mice further suffered more extensive ulceration and necrosis than TLR2^−/−^ mice (extent of necrosis in colon 2.5 vs 1.3, p<0.01) (Figure 3B, D). Instead, TLR2^−/−^ mice showed signs of regeneration with mucin depletion, enlarged and hyperchromatic nuclei, and increased nuclear to cytoplasmic ratio, all indicative of aberrant crypt foci (ACF), which are known to be pre-neoplastic [36] (Figure 3D, right panels). Furthermore, we observed similar proliferation but reduced apoptosis in TLR2^−/−^ ACF compared to WT ACF (Figure 3E, F), which may contribute to survival of transformed cells. Indeed, quantitative analysis of ACF revealed a significant increase in ACF in TLR2^−/−^ mice compared to WT mice in proximal (p<0.001) as well as distal colons (p<0.01) (Figure 3C).

### TLR2-deficiency have increased cell proliferation and reduced apoptosis during early CAC development

**Figure 4:**
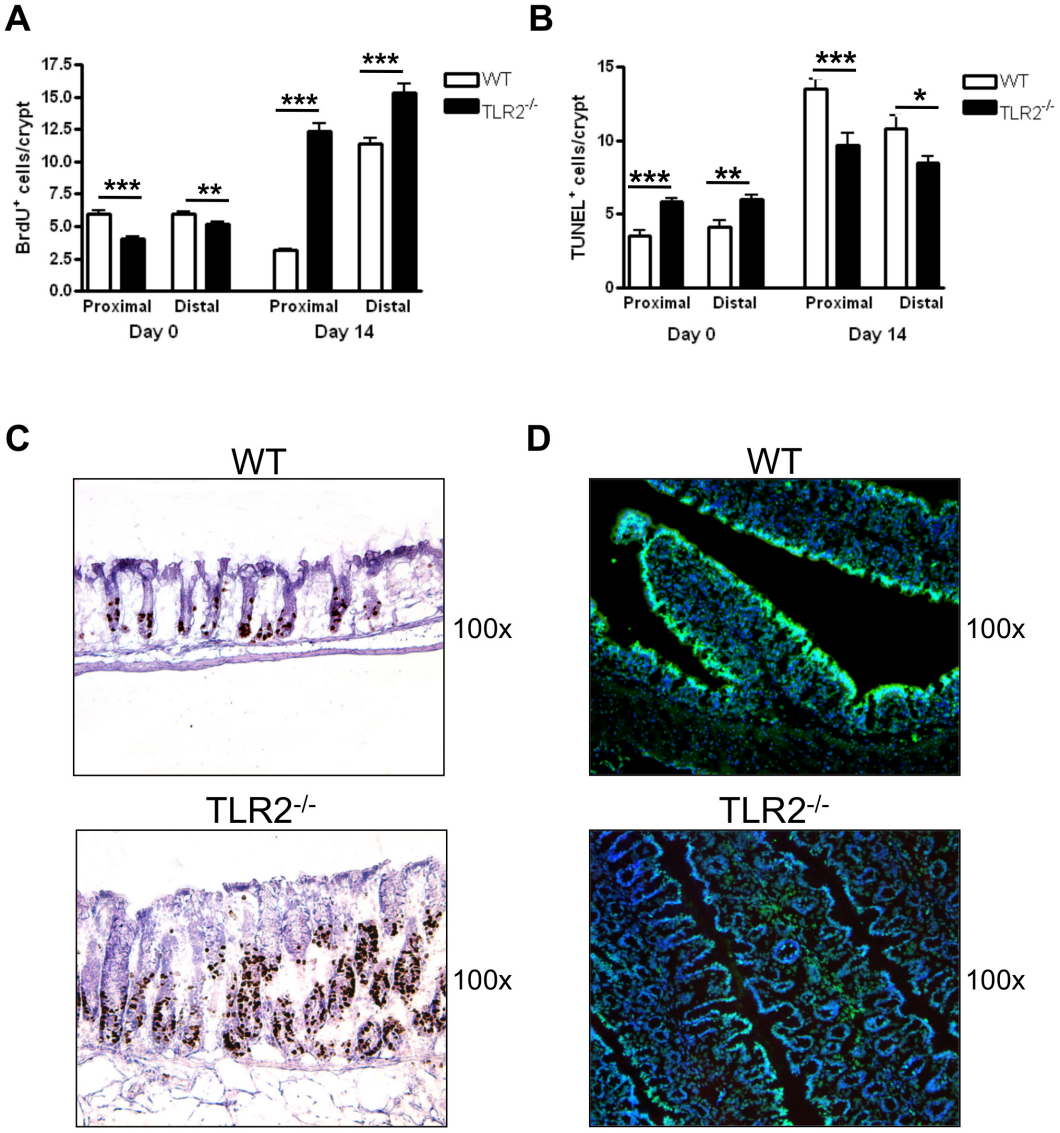
Increased cell proliferation and reduced apoptosis in TLR2-deficient colons during early CAC development. Assessment of proliferation by BrdU staining and apoptosis by TUNEL staining in proximal and distal colons from mice either treated for 14 days with the AOM-DSS regimen (Day 14) or untreated mice (Day 0) (n = 5), BrdU^+^ (A) and TUNEL^+^ (B) cells were counted in intact and well-oriented crypts. BrdU^+^ cells were quantified from at least 20 crypts per region from 4 different slides per animal. (C) Day 14 representative immunohistochemical stains for BrdU in colonic sections. Original magnification 100x. (D) Day 14 representative immunofluorescent stains for TUNEL stains in colonic sections. Original magnification 100x. All tests were performed using 95% confidence intervals. Data are expressed as means ± SEM. * = p<0.05, ** = p<0.01, *** = p<0.001.

Previous studies have demonstrated that recognition of commensal bacteria via TLRs is required for intestinal epithelial homeostasis, which is controlled by the balance of proliferation and apoptosis in the crypts [10]. In order to compare intestinal homeostasis between TLR2^−/−^ mice and WT mice, we examined epithelial cell proliferation by BrdU incorporation and apoptosis by TUNEL staining for DNA fragmentation in colonic crypts. Interestingly, prior to AOM-DSS we observed significantly reduced numbers of BrdU^+^ cells (Figure 4A) with significantly increased numbers of TUNEL^+^ cells (Figure 4B) per crypt in the proximal and distal colons of TLR2^−/−^ mice compared to WT mice. In contrast, after one round of DSS treatment (day 14), TLR2^−/−^ colonic crypts displayed increased BrdU^+^ cells (p<0.001) (Figure 4A, C) and decreased TUNEL^+^ cells (p<0.001) (Figure 4B, D) compared to WT mice, indicating dysregulated epithelial homeostasis in TLR2^−/−^ colons during inflammatory conditions. Furthermore, although the BrdU^+^ cells were primarily localized to the stem cell zone of WT crypts, proliferating cells were found extended into the middle regions of the TLR2^−/−^ crypts, in which epithelial cells are normally differentiated and non-proliferating (Figure 4C). Therefore, as mucosal integrity was compromised in colons deficient of TLR2, the ability of the IEC to adhere to appropriate survival and death signals was compromised, resulting instead in dysregulated cell survival. To confirm that early intestinal tumorigenesis in TLR2^−/−^ mice is dependent on DSS-induced inflammation we examined WT and TLR2^−/−^ mice 14 days after a single injection of AOM (no DSS treatment) as controls. We did not observe any discernible ACF or increased inflammation in WT or TLR2-deficient colons in these control experiments (Figure S2) suggesting the importance of inflammation for the development of tumors in the context of TLR2-deficiency.

### TLR2^−/−^ mice have increased IL-6 and STAT3 activation during early intestinal tumorigenesis

**Figure 5:**
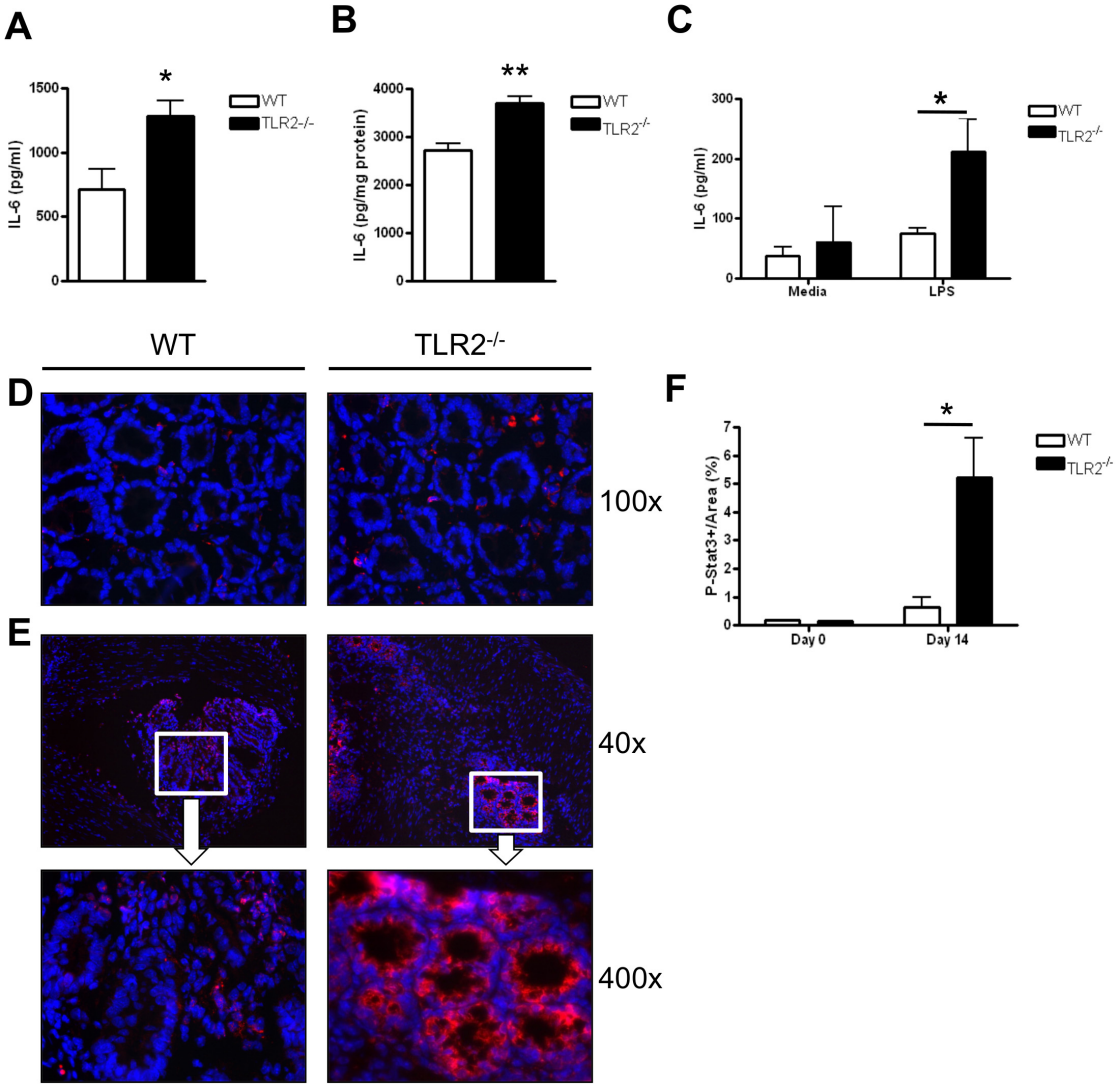
TLR2^−/−^ mice have increased IL-6 and STAT3 activation during early intestinal tumorigenesis. Panels A–C: IL-6 production in WT and TLR2^−/−^ mice at day 14 of AOM-DSS treatment was measured by ELISA. (A) Serum levels of IL-6 (n = 8 WT, n = 9 TLR2^−/−^). (B) IL-6 concentration in colon homogenates was normalized to concentration of protein in the tissues (n = 3 WT, n = 5 TLR2^−/−^). (C) IL-6 secretion from isolated colonic lamina propria cells treated with LPS (1 µg/ml) for 6 h (n = 3–5). (D) Immunofluorescent stains of phospho-Stat3 in colonic tissues of WT and TLR2^−/−^ mice at baseline. Original magnification 100x. (E) Immunofluorescent staining of phospho-Stat3 in colonic tissues of WT and TLR2^−/−^ mice treated for 14 days with the AOM-DSS regimen. Top panel original magnification 40x, bottom panel original magnification 400x. (F) Quantification of phospho-Stat3 intensity measured in ACF from greater than five focus fields in at least four slides per animal (n = 5 WT and n = 5 TLR2^−/−^). All tests were performed using 95% confidence intervals. Data are expressed as means ± SEM. * = p<0.05, ** = p<0.01, *** = p<0.001.

In addition to increased cell proliferation and inflammation, we detected significantly increased serum levels of IL-6 in TLR2^−/−^ mice at early stages of tumorigenesis after initial AOM-DSS treatment (day 14) compared to WT mice (p<0.05) (Figure 5A). Importantly, IL-6 promotes tumor progression in inflammation-associated tumor models through the activation of STAT3 [37], [38]. We did not observe any significant differences in serum concentrations of other pro-inflammatory cytokines or chemokines (IL-12p40, IL-1beta, MCP-1, KC, and MIP-2) between WT and TLR2^−/−^ mice at day 14 of AOM-DSS treatment (data not shown). We also detected increased IL-6 production in whole colon homogenates from TLR2^−/−^ mice at day 14 of AOM-DSS treatment as compared to WT mice (p<0.01) (Figure 5B). Lamina propria mononuclear cells (LPMC) isolated from TLR2^−/−^ mice treated with LPS also produced more IL-6 than WT LPMC (p<0.05) (Figure 5C). Consistent with the increase in IL-6 production in TLR2^−/−^ mice at early stages of tumorigenesis, we observed significantly increased nuclear accumulation of phosphorylated (activated) STAT3 (pSTAT3) in TLR2^−/−^ epithelium compared to WT colons (Figure 5E). Quantification of the intensity of nuclear pSTAT3 in ACF revealed an 8-fold increase in pSTAT3 expression in TLR2^−/−^ ACF compared to WT ACF on day 14 (p<0.05) (Figure 5F). Additionally, while nuclear pSTAT3 expression within TLR2^−/−^ ACF was confined to intact crypt epithelial cells (Figure 5E, right panel), nuclear pSTAT3 in WT ACF appeared mostly in lamina propria or abscessed crypts (Figure 5E, left panel). Quantitative analysis of pSTAT3 at baseline did not reveal any differences in TLR2^−/−^ vs. WT mice (Figure 5F).

### TLR2^−/−^ mice have an increased T_H_17 immune response during CAC development

**Figure 6:**
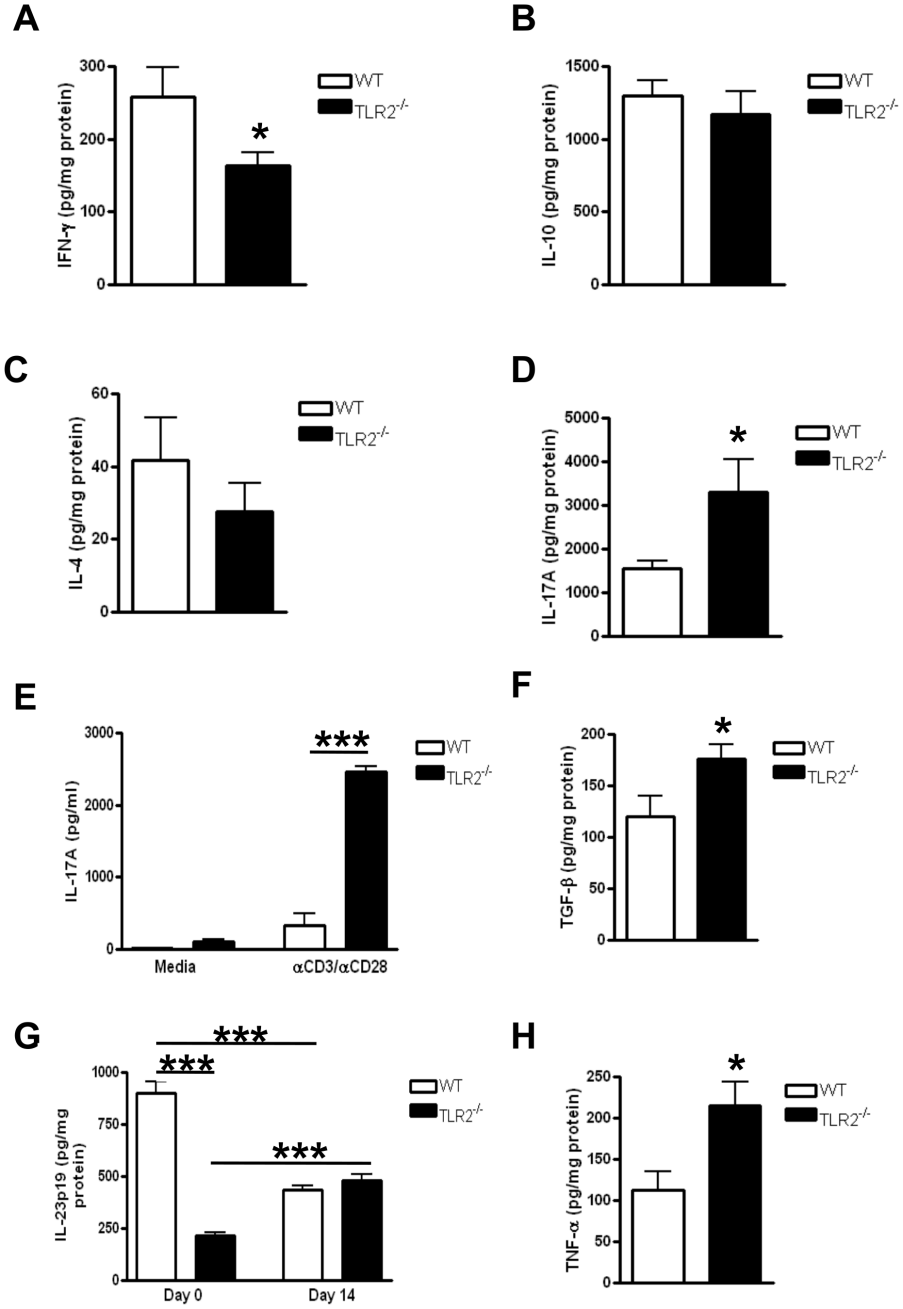
TLR2-deficient mice develop increased T_H_17 responses during early CAC development. (A–D, F–H) Cytokines concentrations in colon homogenates in WT and TLR2^−/−^ mice at day 14 of AOM-DSS treatment were measured by ELISA. (A) IFN-g (n = 5–8). (B) IL-10 (n = 4). (C) IL-4 (n = 4–5). (D) IL-17A (n = 6). (E) IL-17A secretion from isolated chronic lamina propria cells treated with anti-CD3 (1 µg/ml) and anti-CD28 (1 µg/ml) for 72 h (n = 4). (F) TGF-ß (n = 4–10). (G) IL-23p19 (Day 0: n = 8–10; Day 14: n = 4 = 10). (H) TNFa (n = 4–10). All tests were performed using 95% confidence intervals. Data are expressed as means ± SEM. * = p<0.05, ** = p<0.01, *** = p<0.001.

Recent studies have highlighted T_H_17 cells in the pathogenesis of IBD [39] and colon tumorigenesis [40]. We examined the expression of T_H_1, T_H_2 and T_H_17 cytokines in colons of WT and TLR2^−/−^ mice during early development of CAC. While we observed decreased production of IFNg from colon homogenates derived from TLR2^−/−^ mice compared to WT mice on day 14 (Figure 6A), we observed significantly higher TNFa production in TLR2-deficient colons compared to WT colons (p<0.05) (Fig 6H), a Th1 cytokine known to play a crucial role in CAC development [32]. We did not observe any statistically significant differences in IL-10 (Figure 6B) or IL-4 (Figure 6C) levels in the colonic homogenates in these animals. In contrast, we observed a more than 2-fold increase in IL-17A in TLR2^−/−^ colon homogenates compared to WT (p<0.05) (Figure 6D). Isolated LPMCs from TLR2^−/−^ mice also produced greater than 7-fold more IL-17A compared to WT cells when restimulated with anti-CD3e and anti-CD28 (p<0.001) (Figure 6E). We also observed significantly increased production of TGF-b in TLR2-deficient colons compared to WT colons (p<0.05) (Figure 6F). Increased production of TGF-b‥IL-6, and pStat3 in TLR2^−/−^ mice compared to WT further supports the involvement of T_H_17 cells. IL-23p19 is a cytokine that has been implicated in the maintenance of T_H_17 cells [41]. We observed that TLR2^−/−^ colon homogenates have significantly lower IL-23p19 levels at baseline compared to WT colons (p<0.001) (Figure 6G). However, after day 14 of AOM-DSS treatment, IL-23p19 production increased significantly in TLR2^−/−^ colons while decreasing in WT colons (p<0.001) (Figure 6G), further supporting the interplay of TLR2 and T_H_17 cells in this model. Additionally, compared to WT colons at day 14 of AOM-DSS treatment, TLR2^−/−^ colons displayed significantly more colonic TNFa production (Figure 6H), known to play a crucial role in CAC development [32]. Taken together, our data support that TLR2^−/−^ mice following AOM-DSS treatment are skewed towards T_H_17 responses during early CAC development.

### Colonic tissue from TLR2^−/−^ mice recruit inflammatory cells that have reduced NO production and fail to mount an adequate defense against tumor growth

**Figure 7:**
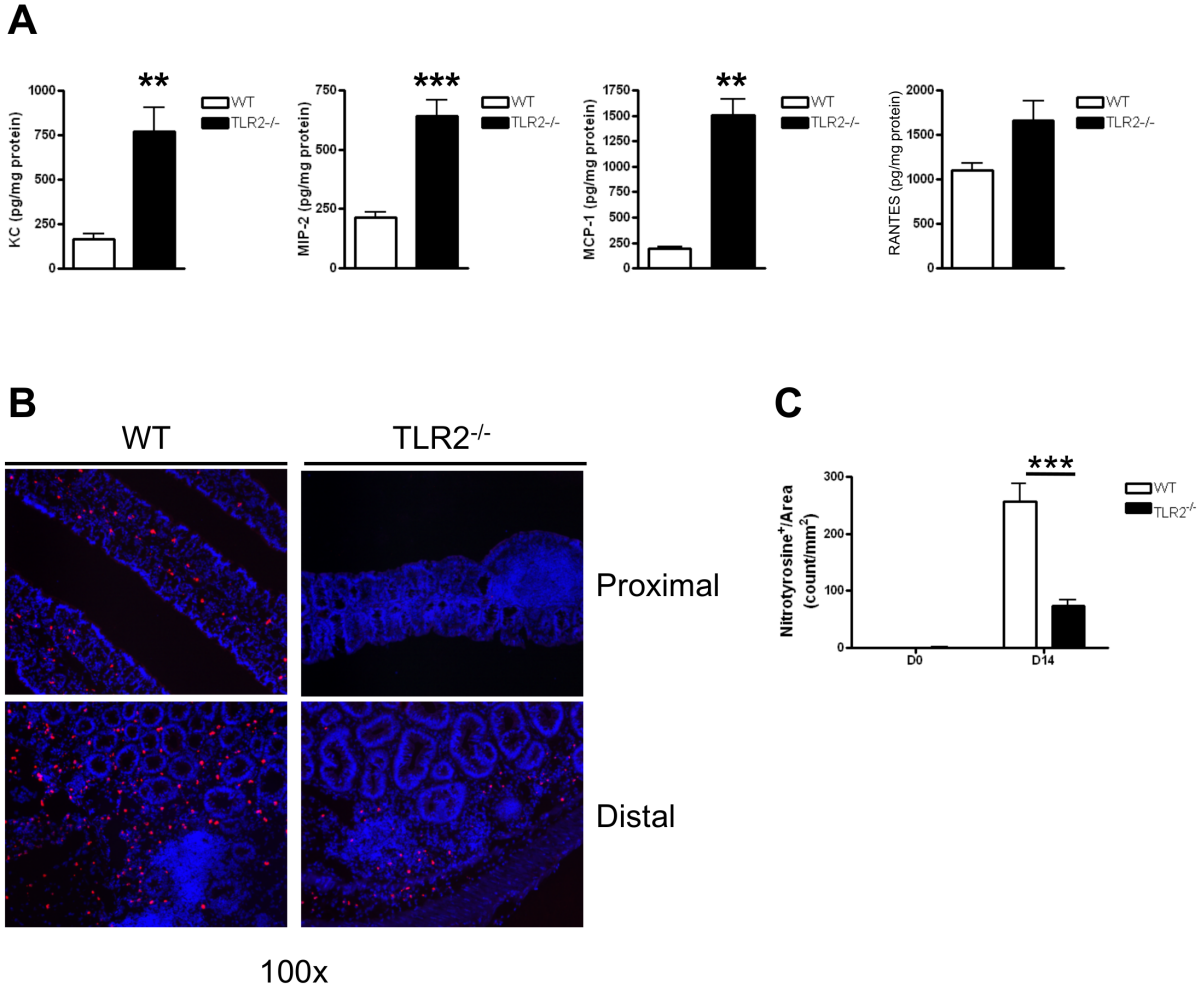
Colonic tissue from TLR2^−/−^ mice recruit inflammatory cells that have reduced NO production and fail to mount an adequate defense against tumor growth in early CAC. (A) Chemokines concentrations quantified by ELISA in colon homogenates in WT and TLR2^−/−^ mice at day 14 of AOM-DSS treatment were measured by ELISA. KC (n = 6 WT, n = 9 TLR2−/−), MIP-2 (n = 6 WT, n = 9 TLR2−/−), MCP-1 (n = 6 WT, n = 4 TLR2−/−), RANTES (n = 4 WT, n = 10 TLR2−/−). (B) Immunofluorescent staining for nitrotyrosine in proximal (top panels) and distal (bottom panels) colon section of WT (left panels) and TLR2^−/−^ (right panels) mice treated for 14 days with the AOM-DSS regimen. Original magnification 100x. (C) Quantification of nitrotyrosine positive cells normalized by area (mm^2^) from greater than five focus fields in at least four slides per animal (n = 5 WT and n = 5 TLR2^−/−^).

To further understand the inflammatory milieu in the colons of mice during early tumorigenesis in our experimental model, we measured the expression of various chemokines in the colonic tissue, including MCP-1 (CCL2), KC (CXCL1), MIP-2 (CXCL2) and RANTES (CCL5), at day 14 of AOM-DSS treatment. Colonic tissues from TLR2^−/−^ mice produced significantly more chemokines that play an important role in the recruitment of neutrophils (KC and MIP-2) and macrophages (RANTES and MCP-1) as compared to WT mice (p<0.01) (Figure 7A,C). These chemokines resulted in increased colonic leukocyte infiltration and overall inflammatory score (Figure 3A). These recruited cells can have either pro- or anti-tumor function. In the presence of increased TGFß they can have a pro-tumor effect and can promote metastases [42], [43]. Since we observed an increase in colonic TGFß production in TLR2^−/−^ mice together with an increase granulocyte/myeloid cell infiltration, we suspected that this recruited inflammatory cell population was conditioned towards a pro-tumor phenotype rather than anti-tumor phenotype. One hallmark of anti-tumor cell infiltrates is increased nitric oxide (NO) production by these cells, which enhances tumoricidal activity, while pro-tumor infiltrates are poor producers of NO [44], [45], [46]. To further investigate the phenotype of these inflammatory cells, we performed immunofluorescent staining to identify nitrotyrosine positive cells, a good biomarker for NO production [47], [48], [49], within lamina propria and near ACF of colons at day 0 and 14 of AOM-DSS treatment (Figure 7B, C). We observed very few nitrotyrosine positive cells in colons of TLR2^−/−^ and WT mice at baseline (Figure 7C). However, by day 14, nitrotyrosine positive cells had infiltrated both proximal (Figure 7B, top) and distal (Figure 7B, bottom) colons, and we observed significantly less nitrotyrosine positive cells in the TLR2^−/−^ colons compared to WT colons (p<0.001) (Figure 7B, C). The reduction in nitrotyrosine positive cells provides evidence that while TLR2^−/−^ mice display increased inflammatory cell infiltrates during early tumorigenesis, these recruited immune cells fail to mount an adequate defense against growing tumors.

## Discussion

Innate immunity, especially Toll-like receptors, plays a significant role in intestinal homeostasis. There is emerging data on the importance of individual components that comprise innate immunity and their relationship to IBD and cancer, however there is a paucity of information on the role of TLR2. We provide a previously unrecognized link between TLR2 and intestinal tumorigenesis under chronic inflammatory conditions. In the absence of TLR2, we observed greater tumor incidence, tumor numbers and tumor sizes in a model of colitis-associated colon cancer, providing evidence for the critical role TLR2 plays in protection from CAC development and progression. In our model, we observed increased inflammation as measured by increased immune infiltration and production of proinflammatory cytokines in TLR2^−/−^ mice, which correlated with the increase in tumor numbers and tumor sizes, in support of previously established links between inflammation and colonic tumorigenesis [30]. We establish a protective role for TLR2 in epithelial injury by (1) maintenance of epithelial homeostasis, (2) maintenance of regulatory mechanisms in colitis-associated inflammation, and (3) development and progression of CAC.

Rakoff-Nahoum et al investigated the role of MyD88 signaling during CAC formation and observed that MyD88−/− mice had reduced tumor numbers [23]. However, that study did not use the AOM-DSS model but used a different experimental model of tumorgenesis, the Apc^Min/+^ model [23]. Additionally, MyD88, which is also critical for proper repair of the intestinal tract, is involved in many critical signaling pathways. Another study conducted by Fukata et al investigated the role of TLR4 during CAC development [24]. They also found reduced numbers of tumors in TLR4−/− mice using the same AOM-DSS model we used in the current study. This study suggests the critical role of TLR4. Possibly TLR4 is the required proinflammatory signaling pathway for CAC development while the data presented here indicates TLR2 provides a suppressive environment in the intestine. In our model, the increase in tumor multiplicity in TLR2-deficient mice, particularly in the proximal colon where there is the highest concentration of TLR2 and bacteria, indicates that a pivotal source of inhibition during inflammation is withdrawn when TLR2 is not present. This allows for dysregulated proliferation and a subsequent increase in CAC tumor development.

Recent studies have shown that intact NFkB signaling is important for tumor development [30], [50]. Further, several studies have linked IL-6 production with colorectal cancer (reviewed in [37]) and levels of serum IL-6 are increased in colorectal carcinoma patients [51], which further correlated with tumor size [52]. In this CAC model, we observed more IL-6 produced in the serum, colon homogenates and lamina propria cells of TLR2^−/−^ mice compared to WT mice. The proliferative and survival effects of IL-6 on IEC are largely mediated by the transcription factor Stat3 [38] as mice lacking Stat3 in colonic epithelium develop fewer adenomas in spite of the fact that they have more severe colitis following exposure to AOM-DSS [38]. In our study we have observed that activated Stat3 was increased within the ACF epithelium of TLR2^−/−^ mice as compared to WT ACF epithelium. IL-6, activated Stat3 and NFkB are intertwined in the development of CAC. Not only can Stat3 increase NFkB activity in tumors, but activation of Stat3 in immune cells in the tumor is dependent on NFkB and IL-6 which is downstream from NFkB. Interestingly, activated Stat3 inhibits lkB kinases (IKKs) thus reducing NFkB associated T_H_1 immunity [53]. We observed that mice deficient in TLR2 produced significantly less IFNg and more TGFß, which coupled with the increase in IL-6 production, led us to a T_H_17 skew. Indeed, IL-17 is a downstream target of IL-6-induced Stat3 signaling [38], [54], [55], [56]. The T_H_17 skewing that we have observed in TLR2-deficient mice in the context of colitis-induction in this study is consistent with recent findings linking IL-17 and IL-23 variants with increased susceptibility to IBD [57], [58]. Several animal models of colitis-induced colorectal cancer have already linked IL-17 production with increased tumorigenesis [40], [59] and epithelial protection in the face of colitis [60]. Additionally, Chae et al recently showed that in the Apc(Min/+) mouse model, IL-17A−/− mice had drastically reduced intestinal tumor numbers [61]. Hence, activation of Stat3 within the ACF epithelium of TLR2^−/−^ mice appears to promote a rise in NFkB driven pro-cancer T_H_17 inflammatory pathway while suppressing T_H_1 immunity.

We observed an increase in inflammatory infiltrates in TLR2^−/−^ colons, consistent with prior studies that used DSS alone and found that TLR2 deficiency may lead to exacerbation of intestinal inflammation (10) and that TLR2 controls and protects mucosal inflammation by regulating tight-junction (TJ) associated barrier integrity [8]. However, as in the AOM/DSS model, we observed a decrease in crypt damage in TLR2 deficient colons as compared to WT colons. Further, consistent with the increase in anti-apoptotic ACFs detected in TLR2-deficient, TLR2^−/−^ crypts showed less cryptitis and ulceration and displayed more signs of regeneration. Conversely, WT colons exhibited more crypt atrophy and regions of ulceration. Thus, in TLR2-deficient mice, tumor inducing circumstances, such as AOM/DSS, may suppress crypt damage and promote proliferation by cytokines such as TGF-ß.

A recent study by Boulard et al [62] investigated the role of TLR-2 in IBD where they used both a T-cell transfer model, and a *H. hepaticus* model, to investigate the innate and adaptive responses during the induction of chronic colonic inflammation. They found that TLR2 played little to no role in the induction of either innate or adaptive responses during the development of the inflammation. The authors concluded that the role of TLR-2 in IBD might differ depending on the model and previous reports indicated a requirement for TLR2 during acute intestinal inflammation models such as DSS used here in this study [8], [10]. Whether there is any difference in the involvement of TLR2 in acute versus chronic models of intestinal inflammation and CAC development is unknown at this time.

The role of TLR2 in colitis-associated colorectal cancer development is not well understood. Our study reveals a protective role for TLR2 in this process. In this study we show *in vivo* evidence that TLR2 signaling during colonic inflammation is essential in regulating proliferation and apoptosis. Based on our observations that TLR2-deficient mice exhibit unregulated proliferative growth and ignorance of apoptotic signals during colitis, we describe a new and critical role for TLR2-induced protection from epithelial transformation. In the genetically susceptible host, IBD results from a breakdown at the epithelial barrier, followed by inappropriate responses to microbial products. TLR2-commensal signaling provides enhanced epithelial barrier protection and pro-survival signals to intestinal epithelial cells especially in the context of inflammation by playing a critical role in promoting regulatory immune responses and preventing pro-tumor inflammatory skewing, thereby providing a protective role in the development and progression of colitis-associated colorectal cancer. These findings have broad implications towards the pathogenesis as well as treatment of colitis-associated colorectal cancer in patients with IBD.

Future studies similar to those that reported the essential role of epithelial expression of TLR4 impacting expression of proinflammatory mediators within the lamina propria [25] utilizing bone marrow chimeras will help dissect the importance of TLR2 in each compartment of intestinal immunity. Also, since supplementation with TLR2 ligands during DSS-induced colitis is shown to promote transepithelial resistance and thereby protect from colitis [8], treatment with TLR2 ligands during the full course of AOM-DSS could result in a reduction of tumor development. These observations could lead to new exciting prophylactic and treatment approaches for colitis-associated colorectal cancer.

## Supporting Information

Figure S1 TLR2-deficient mice develop more progressive colorectal tumors. Numbers of carcinoma in situ per mouse induced by AOM-DSS treatment at day 61 (n = 19 for WT and n = 21 for TLR2-/- mice). The data shown are means ± SEM. Welch's T-test, p = 0.19.
(0.27 MB TIF)

Figure S2 Treatment with AOM alone does not induce early ACF. Representative H&E stains of proximal (A–B) and distal (C–D) colons from WT and TLR2−/− mice 14 days after injection of AOM without DSS treatment (n = 5 for WT and TLR2−/−, respectively). (A) Original magnification 20 x. (B) Original magnification 100 x. (C) Original magnification 40 x. (D) Original magnification 400 x.
(6.70 MB TIF)
