# Supplementary figures and images for: Toll-Like Receptor 2 Signaling Protects Mice from Tumor Development in a Mouse Model of Colitis-Induced Cancer
Source: PLoS One. 2010 Sep 27;5(9):e13027. doi: 10.1371/journal.pone.0013027 (PMC2946405; doi:10.1371/journal.pone.0013027)

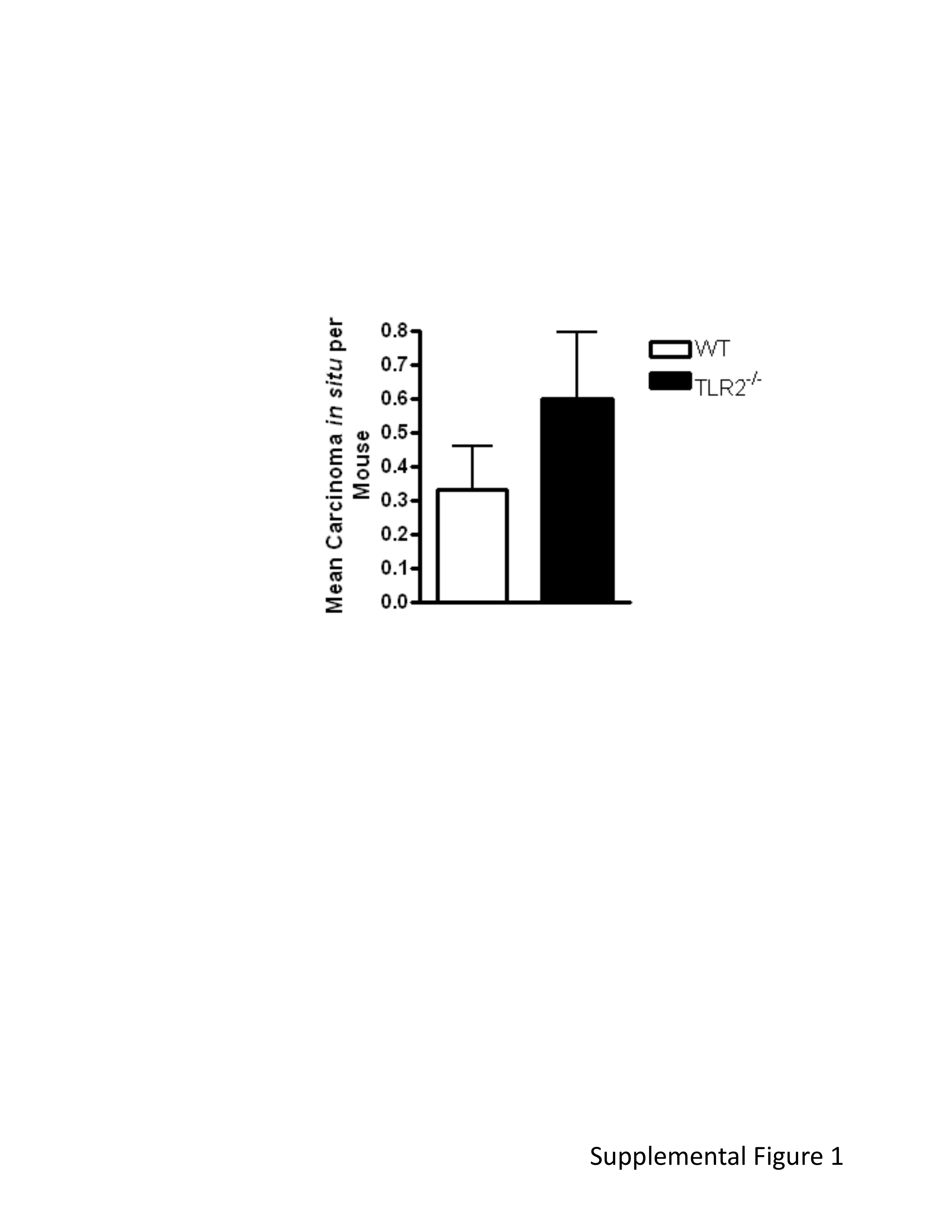

Supplement: Figure S1 — TLR2-deficient mice develop more progressive colorectal tumors. Numbers of carcinoma in situ per mouse induced by AOM-DSS treatment at day 61 (n = 19 for WT and n = 21 for TLR2-/- mice). The data shown are means ± SEM. Welch's T-test, p = 0.19. (0.27 MB TIF) [file pone.0013027.s001.tif]

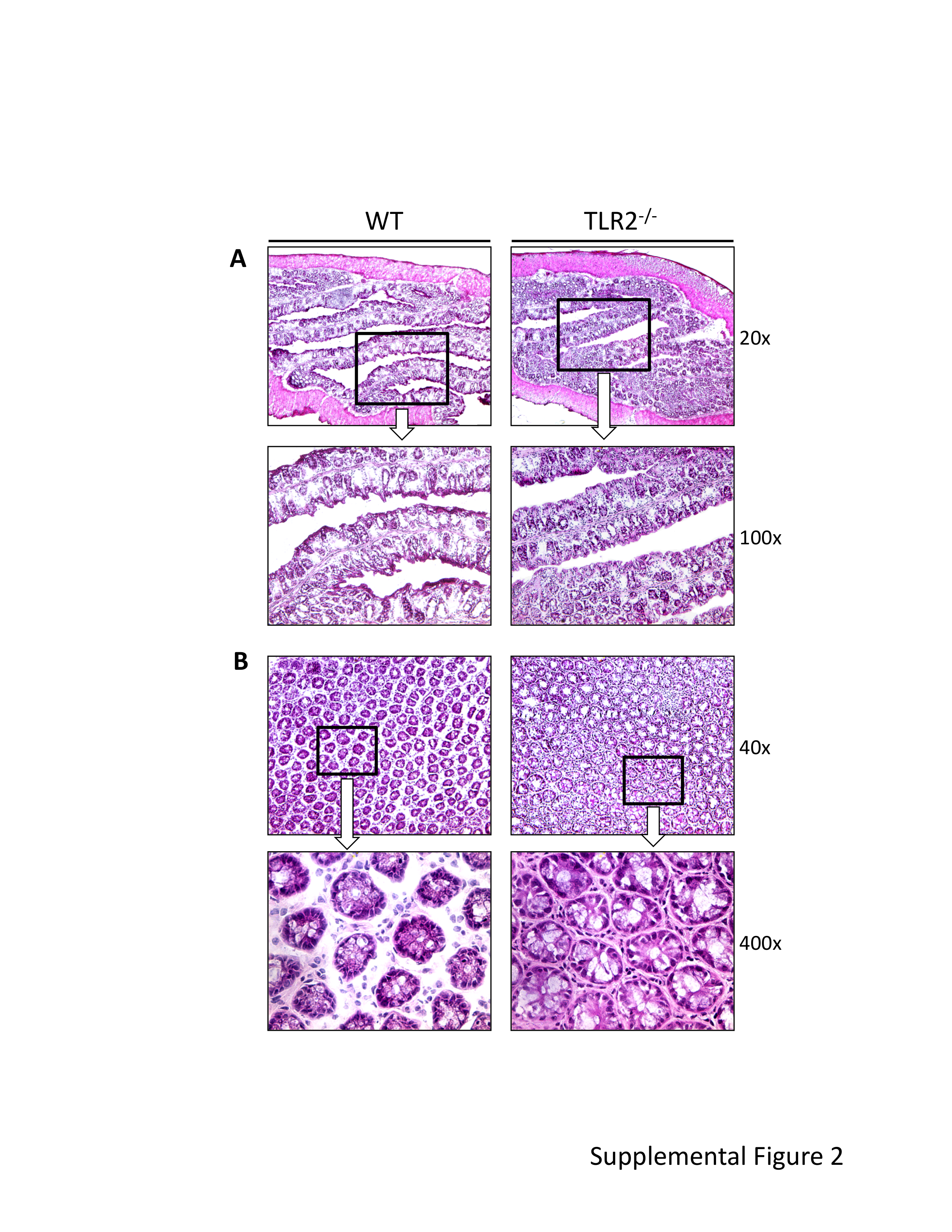

Supplement: Figure S2 — Treatment with AOM alone does not induce early ACF. Representative H&E stains of proximal (A–B) and distal (C–D) colons from WT and TLR2−/− mice 14 days after injection of AOM without DSS treatment (n = 5 for WT and TLR2−/−, respectively). (A) Original magnification 20 x. (B) Original magnification 100 x. (C) Original magnification 40 x. (D) Original magnification 400 x. (6.70 MB TIF) [file pone.0013027.s002.tif]
